# Neural bases of reward anticipation in healthy individuals with low, mid, and high levels of schizotypy

**DOI:** 10.1038/s41598-023-37103-2

**Published:** 2023-06-19

**Authors:** F. Carruzzo, A. O. Giarratana, L. del Puppo, S. Kaiser, P. N. Tobler, M. Kaliuzhna

**Affiliations:** 1 Clinical and Experimental Psychopathology Laboratory, University Hospital Geneva, Belle-Idée, Bâtiment Les Voirons, Chemin Petit-Bel-Air 2, 1226 Thônex, Switzerland; 2 Laboratory for Social and Neural Systems Research, Department of Economics, University of Zurich, Zurich, Switzerland

**Keywords:** Neuroscience, Motivation, Reward

## Abstract

A growing body of research has placed the ventral striatum at the center of a network of cerebral regions involved in anticipating rewards in healthy controls. However, little is known about the functional connectivity of the ventral striatum associated with reward anticipation in healthy controls. In addition, few studies have investigated reward anticipation in healthy humans with different levels of schizotypy. Here, we investigated reward anticipation in eighty-four healthy individuals (44 females) recruited based on their schizotypy scores. Participants performed a variant of the Monetary Incentive Delay Task while undergoing event-related fMRI.Participants showed the expected decrease in response times for highly rewarded trials compared to non-rewarded trials. Whole-brain activation analyses replicated previous results, including activity in the ventral and dorsal striatum. Whole-brain psycho-physiological interaction analyses of the left and right ventral striatum revealed increased connectivity during reward anticipation with widespread regions in frontal, parietal and occipital cortex as well as the cerebellum and midbrain. Finally, we found no association between schizotypal personality severity and neural activity and cortico-striatal functional connectivity. In line with the motivational, attentional, and motor functions of rewards, our data reveal multifaceted cortico-striatal networks taking part in reward anticipation in healthy individuals. The ventral striatum is connected to regions of the salience, attentional, motor and visual networks during reward anticipation and thereby in a position to orchestrate optimal goal-directed behavior.

## Introduction

Reward processing is a core element of goal-directed behavior. It provides organisms with a probabilistic framework to direct attention and guide behavior towards more valued actions and away from less valuable actions. Reward processing includes a phase of reward anticipation, which precedes the consumption phase, elicited by the reception of reward. Correctly anticipating a reward enables motivated behaviors and adaptively initiating actions. The monetary incentive delay (MID) task^1^ has often been used to assess brain regions related to reward anticipation^2–4^. To elicit reward anticipation, the MID presents participants with cues that inform them about the amount of money they can win per trial. These cues are followed by a simple discrimination task and with a percentage of the cued money amount if they respond accurately in the discrimination task. The MID has been modified by Simon, Cordeiro^5^ to adapt to participants’ mean response time and thereby allow every participant to receive a similar amount of money at the end of the task.

Studies using the MID in healthy participants have identified a network of areas underpinning reward anticipation and preparing an individual for action. Robust brain-wide activations have been shown in the ventral striatum, bilateral insula, cingulate cortex, thalamus, premotor cortex and occipital cortex, amongst others^6,7^. The interplay between these regions is thought to facilitate the detection of salient upcoming events and prepare goal-directed actions^1,8,9^.

However, the functional cortico-striatal networks of reward anticipation in healthy participants remain to be established. While many studies have assessed task-based functional connectivity deficits in pathological populations^4,10,11^, as well as individual differences in healthy participants^12–14^ only one study specifically described mean cortico-striatal functional connectivity in healthy individuals^15^. In their study, Cao, Bennett^15^ demonstrated functional connectivity between the ventral striatum and the supplementary motor area, the dorsal cingulate cortex, the anterior insula and the medial occipital lobe. However, these results come from adolescents and cannot be directly generalized to adults, as adolescents have been shown to process reward anticipation differently than adults^16^.

Interestingly, ventral striatal activity during reward anticipation is lower in patients with schizophrenia than in healthy controls^3,17,18^, and correlates negatively with negative symptoms^2,17,19,20^. In individuals high in schizotypal personality traits (SPT), ventral striatal activity to reward predicting cues seems preserved at the group level^21,22^, although Yan, Wang^22^ found less ventral striatal activity in participants with mainly negative symptoms. Similarly, resting-state functional magnetic resonance imaging (rs-fMRI) studies have shown cortico-striatal functional connectivity disturbances in schizophrenia^23,24^ and in individuals high on SPT^25^.

Our study aimed to replicate previous studies on healthy individuals’ whole-brain activations during reward anticipation. In addition, we sought to describe for the first time cortico-striatal functional connectivity during reward anticipation specifically in healthy adult participants. Having access to schizotypy and negative symptom scores for all of our participants, we performed exploratory analyses on correlations between these scores and whole-brain activity. We also explored potential activity and connectivity differences between participants with high schizotypy and participants with low schizotypy scores. Since very few published studies have investigated reward anticipation deficits in schizotypy, the hypotheses we formulated are speculative. Because schizotypy is thought to lie on a continuum between health and schizophrenia, we expected to find similar results for participants with high schizotypy scores and patients with schizophrenia in terms of activity and functional connectivity.

## Methods and materials

### Participants

We conducted a power analysis for the correlation tests we were planning to perform (for a two-tailed correlation of *ρ* = 0.3, at *α* = 0.05 and *β* = 0.8). This showed that we needed to include 84 participants in our sample. 928 individuals were screened by phone from the general population at the University of Zurich. Amongst the individuals that passed the screening process (i.e. over 18 years old, no history of psychiatric disorders, no history of drug use and MRI compatible) we selected participants based on their SPT scores (10% with the lowest scores, 20% with average scores and 10% with the highest scores). In total, 86 participants were recruited (29 low SPT, 26 mid SPT, and 31 high SPT). Two participants from the mid SPT group were excluded from analyses due to non-processable fMRI data. In total, we analyzed the data of 84 participants (44 females). The project was approved by the Ethics Committee of the Kanton (KEK) of Zurich. All methods were implemented following the relevant guidelines and regulations. All participants provided written informed consent to take part in the study, in accordance with the Declaration of Helsinki.

### Clinical assessment

Every participant was clinically assessed with online questionnaires to evaluate negative symptoms and schizotypal personality traits. Negative symptoms were self-assessed using the Self-evaluation of Negative Symptom scale [SNS^26^]. Schizotypal personality traits were assessed with the Schizotypal Personality Questionnaire [SPQ^27^].

### Experimental design and task

**Figure 1:**
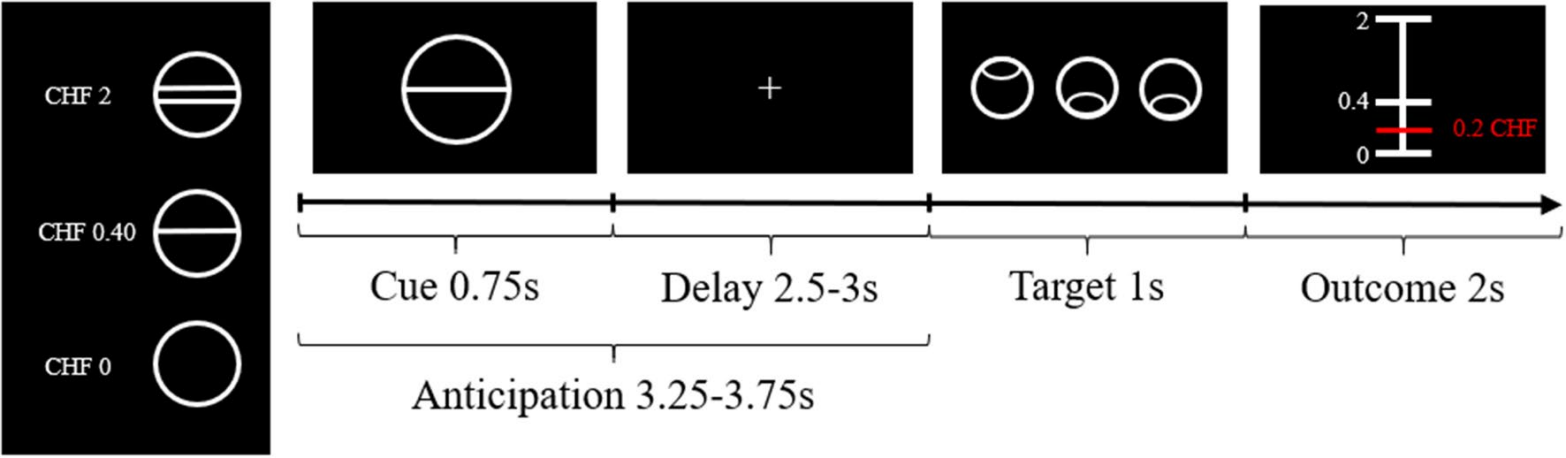
The Monetary Incentive Delay (MID) task. In this task, reward anticipation is modelled as the time between the presentation of the cue and the presentation of the target.

Reward anticipation was assessed using a modified version of the Monetary Inventive Delay task [MID; Fig. 1]^1^ developed by Simon and colleagues^5^ and implemented using the Psychophysics Toolbox^28–30^. At the beginning of each trial, a central cue (0.75 s) indicated the maximum amount of reward winnable (0CHF, 0.40CHF, 2CHF) and was followed by a fixation cross (2.5-3 s). Participants were then asked to discriminate the incongruent target within a set of three circles (until a response was given, 1 s maximum). Next, a feedback screen (2 s) was presented. In case of a correct response, the feedback screen showed the amount of reward won for that trial. If there was no response, the feedback screen asked participants to respond faster. The amount of reward won was calculated as a percentage of the cued amount based on the difference between the response time in the present trial and the mean response time in the previous 15 trials. The mean response time up to the 15th trial was calculated using a pre-defined fixed array of response times from previous piloting [Mrt = 5.95 s, SDrt = 0.66 s; 19]. Finally, the feedback screen was followed by a jittered intertrial interval (ITI, 1 to 9 s, with a mean of 3.5 s).

Every participant completed 12 training trials outside of the scanner to get used to the task and 6 training trials inside the scanner to get used to the MRI environment and the response box. After being informed that they would receive the total amount of money won in the scanner, participants performed two test sessions within the scanner (36 trials each, 12 trials per condition). Each trial lasted about 10 s and each test session lasted about 6 min.

### Behavioral analyses

We performed behavioral analyses using R^31^. Response times were modeled as the time between target presentation and button press. A paired-sample t-test was conducted to assess the difference in response times between high reward trials and no reward trials. We performed a repeated-measures ANOVA with schizotypy classification (high and low) as the between-subject factor and reward (high and no) as the within-subject factor. We performed correlations between reward-related speeding (i.e. the difference between response times for the high reward condition and response times for the no reward condition) and SNS total and apathy scores, as well as SPQ total scores.

### Image acquisition

Our fMRI data was acquired on a Philips Achieva 3.0 T whole-body scanner at the Zurich Center for Neuroeconomics, University of Zurich, with a 32-channel SENSE head coil. Each session consisted of 195 functional images using an echo-planar image (EPI) sequence with 40 slices covering the whole brain acquired in ascending order. The in-plane resolution was 3 mm × 3 mm, 3 mm slice thickness and 0.5 mm gap width over a field of view of 240 mm × 240 mm. The SENSE P reduction (AP) was set to 1.5. Volumes had a repetition time of 2334 ms, an echo time of 30 ms and a flip angle of 90°. The water/fat shift over bandwidth was 13.931 px/31.2 Hz. The first 5 scans were discarded to account for magnetic field equilibration.

We acquired fieldmaps immediately after the MID task. They consisted of 50 slices with an in-plane resolution of 3 mm × 3 mm, 3 mm slice thickness and 0 mm gap over a field of view of 240 mm × 240 mm. There was no SENSE reduction. The repetition time was set to 1150 ms and the echo time to 4.6 ms, with a flip angle of 72°. The water/fat shift over bandwidth was 0.490 px/885.6 Hz.

We acquired anatomical data during the same session, in the same scanner, with the same head coil, using an ultrafast gradient echo-T_1_-weighted sequence in 170 sagittal plane slices of 256 mm × 256 mm resulting in 1 mm × 1 mm × 1 mm voxels. The repetition time was set to 8.3 ms, the echo time to 3.9 ms and the flip angle to 8°. The SENSE P reduction (AP) was set to 1 and the S reduction (RL) was set to 2. The water/fat shift over bandwidth was 2.268 px/191.5 Hz.

### Image preprocessing

We used the Art toolbox (http://web.mit.edu/swg/software.htm) to detect motion and susceptibility artifacts. In total, 0.41% of all scans were outliers (head motion above 2 mm and/or changes in mean signal intensity above 9). The highest percentage of outlier scans for any participant was 7.23%. No participant was excluded after the quality check.

We used SPM12 (Statistical Parametric Mapping, Welcome Trust Centre for Neuroimaging, London, UK) on MATLAB R2019b (Mathworks, Natick, MA, USA) to perform preprocessing and all of our analyses. Preprocessing steps included slice timing correction (the first slice was used as the reference slice), realignment and unwarping with fieldmap correction (with reslicing), coregistration (with reslicing), segmentation, normalization (using forward deformation obtained from segmented images based on tissue probability maps as templates) and smoothing using a 4 mm full-width at half-maximum Gaussian kernel.

### Subject (first)-level models

We modelled first level event-related responses with a general linear model (GLM). The model comprised three regressors for the anticipation phase and three regressors for the consumption phase based on the three conditions of our task (i.e. no reward, low reward, high reward). The consumption regressors for the low and high reward were parametrically modulated with the particular amount of reward received in each trial. We added one regressor modelling target presentation phase, and, if errors trials were present in the session, three regressors modelling anticipation, consumption and target presentation for these trials. The12 regressors in the GLM were convolved with the canonical hemodynamic response function. Reward anticipation was modelled as the contrast between two regressors of the anticipation phase, namely [high reward > no reward]. Six movement parameters were modelled as covariates of no interest and outlier scans discovered by the Art toolbox were added as covariates to be scrubbed. We removed low-frequency noise using a high-pass filter with a cut-off of 0.008 Hz. We also corrected the time-series for serial autocorrelations using an autoregressive AR(1) model.

#### PPI model

Left (lVS, MNI coordinates [x y z] = − 10, 8, − 2; cluster size = 307) and right (rVS, MNI coordinates [x y z] = 20, 16, 0; cluster size = 366) ventral striatum seed regions were defined as the activity cluster within the second level whole-brain probability map masked with meta-analytic ROIs extracted from Neurosynth^32^ using “reward anticipation” as a search term (92 studies, 2913 activations). This approach was selected to render our results as generalizable as possible.

The connectivity maps of lVS and rVS during reward anticipation were assessed separately using a PPI analysis^33^. We defined the psychological factor as the contrast between high and no reward conditions. We then used the PPI toolbox in SPM12 to calculate the interactions between the physiological and psychological factors. We modelled PPI interaction, seed activity and onset regressors of the activation GLM for each seed region in an individual GLM for each participant together with two session constants.

### Whole-brain activity analyses during reward anticipation

#### Whole-brain activation analyses

We performed a whole-brain analysis on reward anticipation [high reward > no reward] using a one-sample t-test (primary threshold of *p* < 0.05 FWE, and a cluster-level threshold of *p* < 0.05 FWE), with all groups taken together. We used a stringent primary threshold of *p* < 0.05 FWE instead of the classical *p* < 0.001 uncorrected due to the highly significant whole-brain one-sample t-test (i.e. large clusters spanning multiple regions). A more stringent threshold was necessary to extract clusters we could clearly define.

We also performed an exploratory whole-brain analysis on the contrast [high reward > low reward] using a one-sample t-test with the same parameters as our main analysis.

#### Regression with negative symptoms and schizotypal personality

We performed regression analyses to assess the relationship between whole-brain activity and total negative symptoms and apathy scores using the SNS and schizotypal personality using the SPQ. To do so, we used one-sample t-tests (primary threshold of *p* < 0.001 uncorrected, and a cluster-level threshold of *p* < 0.05 FWE) with each regressor of interest.

#### Categorical differences between high and low schizotypy groups

Group differences between high SPT and low SPT in whole-brain localized activity were assessed using a two-sample t-test (primary threshold of *p* < 0.001 uncorrected, and a cluster-level threshold of *p* < 0.05 FWE). We also assessed categorical differences in ventral striatal activity between high SPT and low SPT using both rVS and lVS seeds taken together as a mask on a two-sample t-test.

### Psychophysiological interaction analysis

#### Whole-brain functional connectivity analyses

We performed a one-sample t-test on individual connectivity maps based on the first-level reward anticipation contrast [high reward > no reward] using lVS and rVS seeds separately (primary threshold of *p* < 0.001 uncorrected for clusters of more than 30 voxels, and a cluster-level threshold of *p* < 0.05 FWE), analyzing all groups together. Note that this methodology does not allow the localization of the connectivity signal within a cluster.

#### Regression with negative symptoms and schizotypal personality

We assessed the link between cortico-striatal functional connectivity and regressors of interest by performing a one-sample t-test on individual connectivity maps [high reward > no reward] using both lVS and rVS seeds (primary threshold of *p* < 0.001 uncorrected for clusters of more than 30 voxels, and a cluster-level threshold of *p* < 0.05 FWE). Regressors of interest included the SNS total and apathy scores and the total score and negative factor of the SPQ for schizotypal personality.

#### Categorical differences between high and low schizotypy groups

Group differences between high SPT and low SPT in cortico-striatal connectivity during reward anticipation were assessed using a two-sample t-test on individual connectivity maps with lVS and rVS seeds (primary threshold of *p* < 0.001 uncorrected for clusters of more than 30 voxels, and a cluster-level threshold of *p* < 0.05 FWE).

## Results

### Behavioral results

The main characteristics of our sample can be found in Table 1. Behavioral analyses indicated that participants responded to high reward targets (*M*_*high*_ = 0.42, *σ* = 0.07) on average more quickly than to no reward targets (*M*_*no*_ = 0.52, σ = 0.13; *t*(83) = 14.49, *p* < 0.001, *d*_*Cohen*_ = 1.58). However, there were no group differences between participants with high schizotypy and participants with low schizotypy (*F*(58) = − 1.07, *p* = 0.29). Additionally, reward-related speeding did not correlate with SNS total and apathy scores, nor with SPQ total scores (all *ps* > 0.76). In addition, neither SNS total and apathy scores, nor SPQ total scores, correlated with mean Framewise Displacement (all *ps* > 0.43).

**Table 1:** Summary of demographic, psychopathological, and clinical characteristics.

Characteristics	All SPQ	Low SPQ	Mid SPQ	High SPQ
Age (year)	22.44 ± 2.80	22.66 ± 2.91	22.33 ± 2.97	22.32 ± 2.62
Sex (female/total)	44/84	13/29	12/24	15/31
SNS scores				
Apathy score	6.08 ± 5.56	2.76 ± 2.90	4.13 ± 4.57	10.71 ± 5.08
Total score	10.95 ± 8.42	5.69 ± 5.33	8.71 ± 7.54	17.61 ± 7.04
SPQ scores				
Negative factor	0.51 ± 0.12	0.29 ± 0.06	0.50 ± 0.12	0.73 ± 0.10
Positive factor	0.38 ± 0.14	0.23 ± 0.03	0.37 ± 0.05	0.53 ± 0.10
Total	0.46 ± 0.16	0.27 ± 0.03	0.45 ± 0.02	0.65 ± 0.05

*SNS* Self-evaluation of Negative Symptoms scale, *SPQ* Schizotypal Personality Questionnaire.

### Whole-brain activity analyses during reward anticipation

#### Whole-brain activation analyses

**Figure 2:**
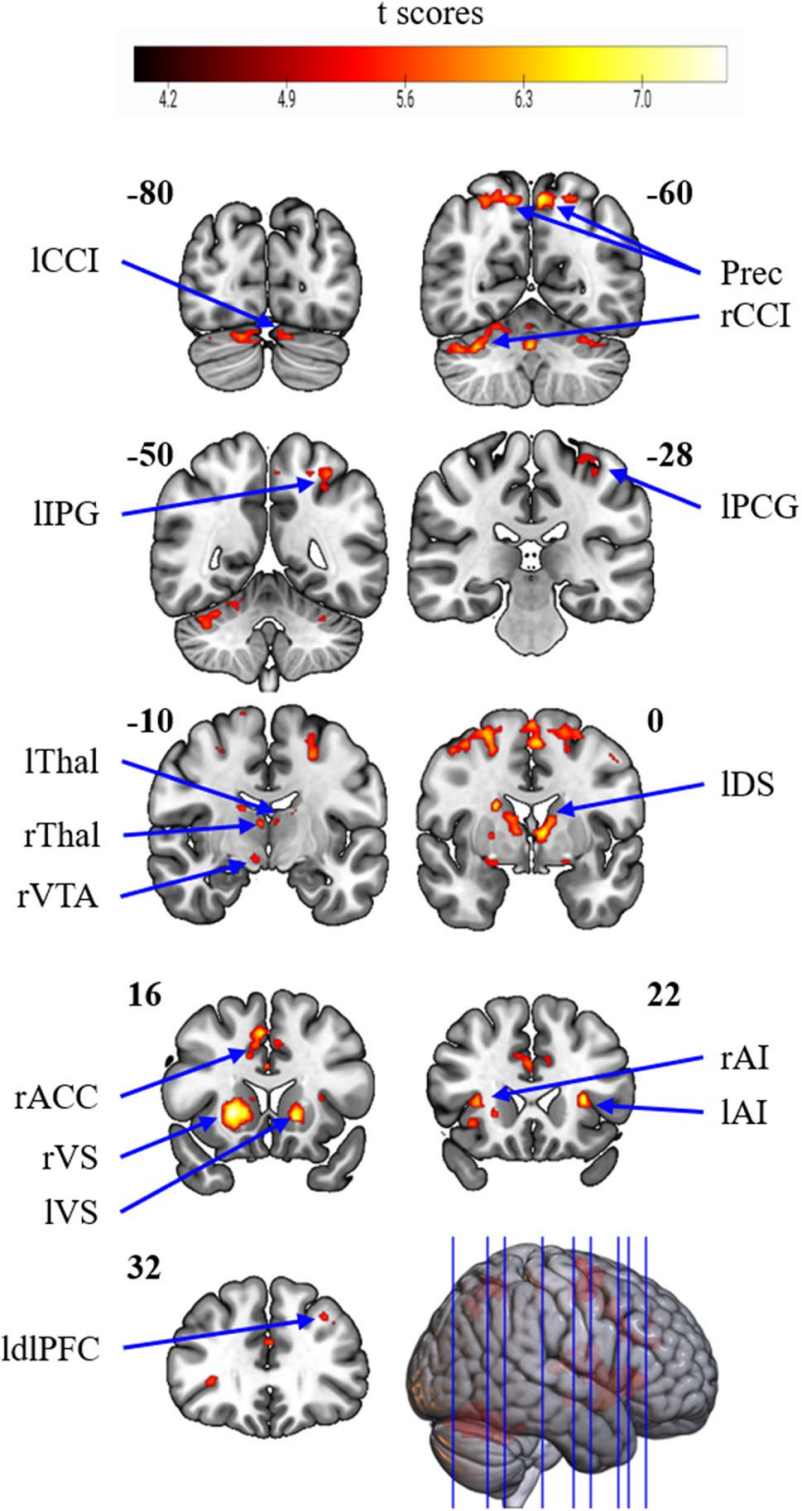
Whole-brain activity analyses (primary threshold of p < 0.05 FWE, and a cluster-level threshold of p < 0.05 FWE) showed activations in the bilateral ventral striatum (VS), dorsal striatum (DS), anterior insula (AI), thalamus (Thal), precuneus (Prec) and cerebellum Crus I (CCI); the right anterior cingulum (ACC), ventral tegmental area (VTA); and the left precentral gyrus (PCG), dorsolateral prefrontal cortex (dlPFC) and inferior parietal gyrus (IPG). y coordinates are indicated in bold. Labelling in this and other figures was done using the Automated Anatomical Labelling Atlas 3^48^.

Whole-brain analyses showed robust reward anticipation activations in the bilateral VS, dorsal striatum, anterior insula (AI), thalamus, precuneus and cerebellum Crus I. Moreover, activity occurred in the right mid cingulum/anterior cingulate cortex, superior frontal gyrus, ventral tegmental area, left precentral gyrus, cerebellum VI, dorsolateral prefrontal cortex and inferior parietal gyrus (Fig. 2, Table 2). Thus, reward anticipation was associated with increased activity in regions processing reward, visual and motor information.

**Table 2:** Whole-brain activation analysis results for the contrast high reward > no reward anticipation.

Conditions	Side	Structures	MNI coordinates			t	Number of voxels
			x	y	z		
High reward > no reward	Bilateral	Subcortical structures	22	16	0	7.53	1452*
		Right ventral striatum	22	16	0		
		Right anterior insula	32	30	2		
		Right dorsal striatum	14	4	14		
		Right thalamus	2	− 20	8		
		Left thalamus	− 4	− 10	12		
		Left ventral striatum	− 16	16	− 2		
		Left dorsal striatum	− 12	0	12		
	Right	Cerebellum crus I	42	− 56	− 30	7.15	865*
		Anterior/mid cingulum	6	14	46	7.13	529*
		Dorsolateral prerontal cortex	24	2	62	6.78	375*
		Ventral tegmental area	8	− 12	− 10	6.54	38*
		Precuneus	12	− 60	56	6.25	160*
		Precuneus	14	− 68	46	6.08	51*
	Left	Precuneus	− 6	− 58	56	7.17	174*
		Anterior insula	− 30	22	8	7.05	77*
		Precentral gyrus	− 48	− 2	48	6.79	39*
		Cerebellum VI	− 30	− 44	− 30	6.59	142*
		Precentral gyrus	− 28	− 2	60	6.30	249*
		Dorsolateral prefrontal cortex	− 32	32	40	6.19	34*
		Inferior parietal gyrus	− 34	− 50	60	6.14	112*
		Cerebellum crus I	− 12	− 80	− 24	5.97	39*
		Precentral gyrus	− 32	− 28	64	5.81	35*

*p < 0.05 FWE corrected at the cluster level for the whole brain (primary threshold: p < 0.05, FWE corrected). Only clusters of 30 voxels or more are presented here.

**Figure 3:**
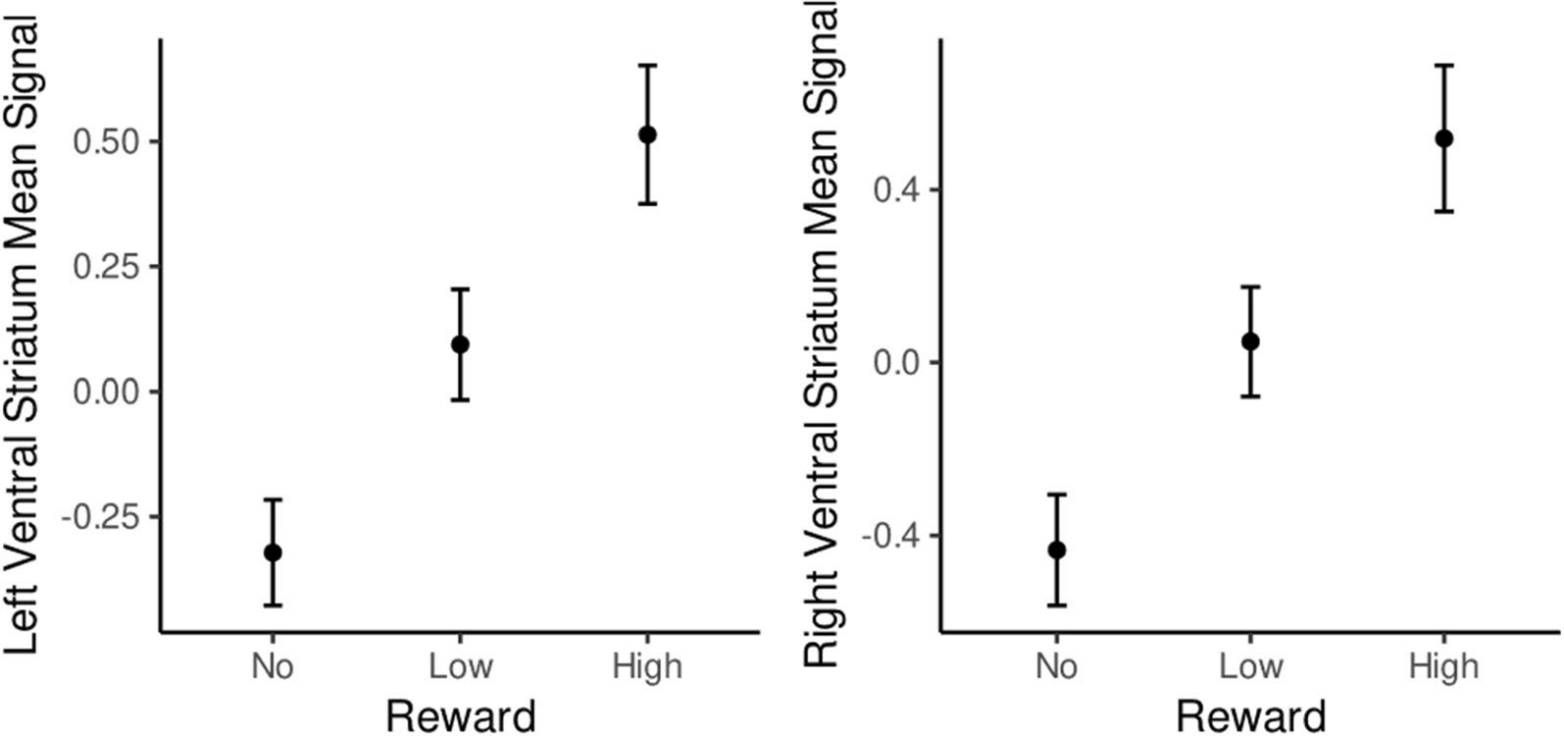
Illustration of the progressive increase of brain activity in response to reward, with the anticipation of higher rewards yielding stronger activation. This illustration is based on the mean signal of the left and right ventral striatum mean signal for the no, low and high reward conditions.

Our exploratory analysis on the contrast [high reward > low reward] showed increased activity in similar cortical and subcortical regions, although effect sizes were smaller than those of the main contrast, as one would expect. To illustrate this, we extracted the activity from the left and right ventral striatum for all three conditions and found that anticipating rewards of increasing magnitude was associated with corresponding increases in BOLD response (Fig. 3).

#### Regression with negative symptoms and schizotypal personality

We found no correlations between whole-brain activity and SNS total and apathy scores, nor with SPQ scores.

#### Categorical differences between high and low schizotypy groups

We found no categorical difference in whole-brain localized activity between high SPT and low SPT groups. Additionally, no categorical difference was found between high SPT and low SPT when looking solely at ventral striatal activity.

### Psychophysiological interaction analysis

#### Whole-brain functional connectivity analyses

**Figure 4:**
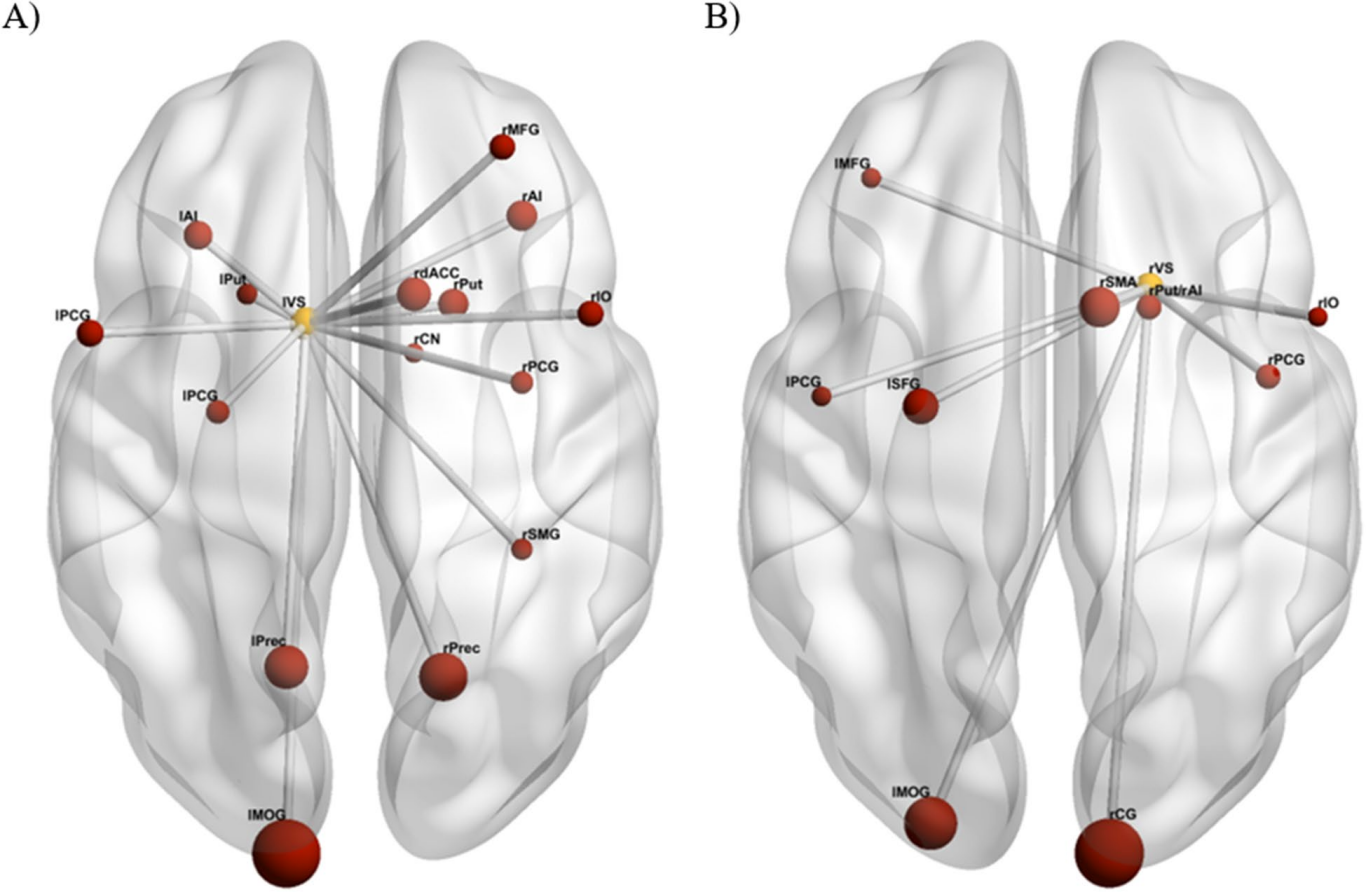
Psychophysiological interaction results (primary threshold of p < 0.01 uncorrected, and a cluster-level threshold of p < 0.05 FWE) for the anticipation phase of the MID task. Whole-brain analysis showed connectivity between the left ventral striatum (lVS) and the bilateral precuneus (Prec), putamen (Put), anterior insula (AI) and precentral gyrus (PCG), the right dorsal anterior cingulate cortex (dACC), mid frontal gyrus (MFG), caudate nucleus (CN), inferior operculum (IO) and supramarginal gyrus (SMG), and the left mid occipital gyrus (MOG). We also found functional connectivity between the right ventral striatum (rVS) and the bilateral precentral gyrus, the right putamen/anterior insula (Put/IA), calcarine gyrus (CG), supplementary motor area (SMA) and inferior operculum, and the left mid occipital gyrus, superior frontal gyrus (SFG) and mid frontal gyrus (MFG). Sphere sizes are based on cluster sizes. Glass brains in this figure were created using BrainNet^49^.

Our analyses showed increased cortico-striatal functional connectivity for high compared to no reward conditions between the lVS and the bilateral precuneus, anterior insula, precentral gyrus, right dorsal anterior cingulate cortex, mid frontal gyrus, caudate nucleus, inferior operculum, supramarginal gyrus, and the left mid occipital gyrus. Additionally, we found increased striato-striatal connectivity between the lVS and the bilateral putamen. We also found stronger reward anticipation-related functional connectivity between the rVS and the bilateral precentral gyrus, the right putamen/anterior insula, calcarine gyrus, supplementary motor area, inferior operculum, the left mid occipital gyrus, superior frontal gyrus and mid frontal gyrus (Fig. 4, Table 3). The ventral striatum therefore showed functional connectivity increases within the reward, saliency, attention and motor networks.

**Table 3:** Whole-brain psychophysiological interaction results for the contrast high reward > no reward anticipation using the lVS and rVS as seeds.

Conditions	Seed	Side	Structures	MNI coordinates			t	Number of Voxels
				x	y	z		
High reward > no reward	lVS	Right	Precuneus	18	− 64	46	5.82	843*
			Putamen	20	12	2	5.47	249*
			Dorsal anterior cingulate cortex	12	14	42	5.16	323*
			Mid frontal gyrus	30	44	34	4.77	167*
			Anterior insula	34	30	− 2	4.73	253*
			Caudate nucleus	12	2	12	4.72	88*
			Precentral gyrus	34	− 4	48	4.56	126*
			Inferior operculum	48	10	30	4.41	171*
			Supramarginal gyrus	34	− 38	40	4.17	109*
		Left	Mid occipital gyrus	− 14	− 100	4	6.36	2797*
			Precuneus	− 14	− 62	50	5.81	633*
			Anterior insula	− 32	26	6	5.25	216*
			Precentral gyrus	− 54	6	30	4.46	188*
			Precentral gyrus	− 28	− 10	52	4.18	135*
			Putamen	− 22	14	− 4	4.15	104*
	rVS	Right	Putamen/anterior insula	20	12	4	5.79	243*
			Calcarine gyrus	12	− 98	4	5.65	1614*
			Supp. motor area	10	12	48	5.07	730*
			Precentral gyrus	44	– 2	48	4.64	237*
			Inferior operculum	54	10	30	4.18	79*
		Left	Mid occipital gyrus	− 24	− 92	2	5.62	1118*
			Superior frontal gyrus	− 26	− 8	58	4.99	578*
			Mid frontal gyrus	− 36	38	24	4.83	90*
			precentral gyrus	− 46	− 6	40	4.40	86*

*p < 0.05 FWE corrected at the cluster level for the whole brain (underlying height threshold: p < 0.001, uncorrected, threshold at 30 voxels). *lVS* left ventral striatum, *rVS* right ventral striatum.

#### Dimensional relationships with negative symptoms and schizotypal personality

No correlations were found between cortico-striatal functional connectivity and SNS total and apathy scores, nor with SPQ scores.

#### Categorical differences between high and low schizotypy groups

We found no categorical difference in cortico-striatal functional connectivity between high SPT and low SPT, with both rVS and lVS seeds.

## Discussion

We designed this fMRI study to assess whole-brain activity and functional connectivity between the ventral striatum and the rest of the brain during reward anticipation in a large sample of healthy individuals. Our analyses showed robust whole-brain activations during reward anticipation. Importantly, our data revealed functional connectivity related to reward anticipation between the ventral striatum and components of the salience, attention, visual and motor networks, in line with the attention enhancing and motor facilitating functions of reward. In addition, we assessed associations between activity and functional connectivity and schizotypal personality and negative symptom scores. Contrary to our hypotheses, we found no correlation between activity or functional connectivity and schizotypal personality and negative symptom scores. Our exploratory analyses also showed no categorical differences between the high and the low schizotypy groups. Taken together, our results suggest that reward anticipation is affected differently in the various stages of the psychosis continuum.

### Whole-brain activity analyses during reward anticipation

Our whole-brain analyses revealed activity in the traditional regions dedicated to reward anticipation, already described by Knutson, Westdorp^1^, comprising the ventral striatum, dorsal striatum and anterior insula. Among these regions, the ventral striatum processes the expected (subjective) value of future rewards and helps compute reward prediction errors^1,8,9^. The dorsal striatum’s role in reward anticipation is the integration of ventral striatal information to select future actions based on the best outcome possible^7,34^. Finally, as a part of the salience network, the anterior insula helps integrate motivational signals with attentional processes^35,36^. The anterior insula also processes outcome uncertainty^37^, which applies to the cues but not the rewards in the modified version of the MID we used.

Additionally, we found activations in the ventral tegmental area (VTA). The VTA is known to have strong connections with the ventral striatum^38,39^ and processes reward prediction errors^40^ and incentive salience^41^ in animal studies. Cortical and cerebellar activations closely matched those described in previous meta-analyses^6,7,36,42^. These included regions dedicated to motor functions, including the primary motor cortex, the supplementary motor area, thalamus and cerebellum^6^, which could facilitate motor preparation when facing highly rewarded trials.

### Cortico-striatal functional connectivity

We observed cortico-striatal networks similar to Cao, Bennett^15^, who described functional connectivity in healthy adolescents. First, we found functional connectivity of the ventral striatum to the salience network, particularly the anterior insula, again suggesting integration of motivation and attention^35,36^. In line with this interpretation, we found connections to attentional network regions such as the supramarginal gyrus and the inferior frontal cortex^43,44^. Increased connectivity with these regions is also compatible with stronger reward anticipation-related communication of the ventral striatum with the visual network, including the calcarine and mid occipital gyrus. Thus, anticipated reward can facilitate visual attention towards reward-predicting cues^45^. We also found connections to motor networks, with the dorsal anterior cingulate cortex and supplementary motor area, indicating once again motor preparation for highly rewarded trials^46^.

Taken together, these results corroborate the well-established notion^47^ that the ventral striatum is at a crossroads of networks that act together to favor rewarded actions over non-rewarded ones. The recruitment of salience and attentional networks during reward anticipation might help disrupt other ongoing processes to focus more specifically on the rewarded stimuli. In contrast, the recruitment of visual and motor networks might prepare humans to perceive and react to rewarded stimuli as fast and accurately as possible.

### No correlation between activity and functional connectivity and schizotypal personality and negative symptom scores

Contrary to our hypothesis, we found no correlation between local activity or functional connectivity and schizotypal personality or negative symptom scores. We also found no difference in local activity or functional connectivity between participants with high schizotypy and participants with low schizotypy. These results converge with those of previous studies reporting unimpaired activity at the group level in participants with comparably high schizotypy scores^21,22^. It is possible that the reward anticipation impairments might appear solely in sub-populations, for example those with high negative schizotypy^22^. However, in our population, the positive and negative factors of the SPQ were highly correlated (r = 0.77) and therefore no selective correlation between whole-brain activity during reward anticipation and negative schizotypy was found.

### Limitations

There are limitations to this study. First, the population we assessed mostly comprised young students, which does not represent the full extent of the variability of people experiencing schizotypy. Additionally, the size of our categorical samples might not be big enough to detect subtle differences in reward anticipation in schizotypy. For example, for a two-tailed t-test, with a strong effect size of 0.7 (based on studies on a comparison between individuals with schizophrenia and healthy controls), an alpha of 0.05, and beta of 0.8, 34 participants are required per group. However, a more modest effect size (which is to be expected given that schizotypy is not a clinical condition and the differences between low and high scorers might therefore be smaller than between patients and controls) significantly increases the required group size. For example, for an effect size of 0.5 (medium), 64 participants are required per group. These limitations could partly explain why we did not find any categorical difference in localized activity and functional connectivity analyses. Additional analyses based on a more extensive population could address these limitations.

### Conclusion

Our analyses confirmed the central role of the ventral striatum during reward anticipation. On the one hand, we replicated previous findings showing activations in the ventral and dorsal striatum, as well as in regions dedicated to salience and motor processing. On the other hand, we identified the functional networks orchestrated by the ventral striatum during reward anticipation in healthy adults. The widespread network of regions interacting with the striatum included components of the salience, attention, visual and motor networks, which conjointly may optimize goal-directed actions. Finally, we showed that reward anticipation might not be equally affected in the psychosis continuum, but instead seems to reflect the gravity of pathology.

## Data Availability

The datasets generated during and/or analysed during the current study are available from the corresponding author on reasonable request.
